# Increased Serum Uric Acid Level Is a Risk Factor for Left Ventricular Hypertrophy but Not Independent of eGFR in Patients with Type 2 Diabetic Kidney Disease

**DOI:** 10.1155/2017/5016093

**Published:** 2017-06-20

**Authors:** Chuchu Zeng, Dongsheng Cheng, Xiaohua Sheng, Guihua Jian, Ying Fan, Yuqiang Chen, Junhui Li, Hongda Bao, Niansong Wang

**Affiliations:** Department of Nephrology, Shanghai Jiao Tong University Affiliated Sixth People's Hospital, 200233 Shanghai, China

## Abstract

**Background:**

Although the relation between serum uric acid (SUA) and left ventricular hypertrophy (LVH) has been studied for decades, however, their association remains debatable.

**Methods:**

This is a retrospective study in which a total of 435 hospitalized Chinese patients with type 2 DKD were enrolled. The subjects were stratified into quartiles according to SUA level. LVH was assessed by two-dimensional guided M-mode echocardiography.

**Results:**

There was a significant increase in the prevalence of LVH in patients with type 2 DKD across SUA quartiles (28.9, 26.5, 36.1, and 49.5%; *p* < 0.001). The Spearman analysis indicated that SUA was positively correlated to LVMI and negatively correlated to eGFR. The logistic regression analysis revealed that the odd ratio for LVH in the highest SUA quartile was 2.439 (95% CI 1.265–4.699; *p* = 0.008; model 1) or 2.576 (95% CI 1.150–5.768; *p* = 0.021; model 2) compared with that in the lowest SUA quartile. However, there was no significant increased risk of LVH in the subjects with the highest SUA quartile after adjusting the eGFR (OR = 1.750; 95% CI 0.685–4.470; *p* = 0.242; model 3).

**Conclusions:**

In selected population, such as type 2 DKD, the elevated SUA level is positively linked with the increased risk of LVH, but this relationship is not independent of eGFR.

## 1. Introduction

Left ventricular hypertrophy (LVH) is an imminent prognostic sign and an independent risk factor for cardiovascular morbidity and mortality [1, 2]. Serum uric acid (SUA), the circulating end product of purine metabolism, is excreted predominantly by the kidney. Accumulated studies indicate that increased SUA level which is associated with endothelial dysfunction, activation of RAAS, increase of oxidative stress, and inflammation may play an important role in the pathogenesis of cardiovascular disease and kidney dysfunction [3–7].

In the recent decades, a number of clinical studies have confirmed the positive correlation between SUA level and LVH in patients suffering from hypertension, cardiac patients, postmenopausal women, or the general population [8–12]. However, in several epidemiological studies, this positive association became vague or did not remain significant after multivariate adjustment for classic risk factors [13, 14]. Thus, although the connection between SUA and LVH has been studied for decades, the relationship remains unclear and debatable.

Therefore, this study aims at the examination of the association of SUA level and LVH in hospitalized Chinese patients with type 2 diabetic kidney disease. To the best of our knowledge, this is the first clinical study to assess the relationship between SUA level and the risk of LVH with special focus on type 2 DKD patients.

## 2. Methods

### 2.1. Population for Study

710 inpatients with type 2 DKD in Shanghai Jiao Tong University Affiliated Sixth People's Hospital were consecutively observed during the period from January 2009 to January 2015. The patients taking diuretics, uric acid-lowering drugs, or other drugs that could interfere with uric acid level were excluded. The patients who did not undergo for echocardiography examination and without complete clinical data were not included. The hemodialysis patients and peritoneal dialysis patients were also not under consideration/under study. As a result, 435 patients, at a mean age of 64 ± 12 years, were the subjects of the current study. The study was approved by the ethical review board of Shanghai Jiao Tong University Affiliated Sixth People's Hospital. All subjects provided their written informed consent for this study.

All subjects underwent an interview and provided their history of hypertension (HTN), coronary heart disease (CHD), cerebral infarction (CI), the use of antihypertensive agents (AHAs) and lipid-lowering drugs (LLDs), and smoking and alcohol consumption habits.

### 2.2. Physical Examination

The body mass index (BMI) was calculated as the body weight divided by the height squared. Blood pressure was measured by a standard mercury magnetometer after the subject had been remained seated for at least 10 min.

### 2.3. Laboratory Assays

Venous blood samples were drawn after an overnight fasting and 2 hours after breakfast. Fasting glycosylated hemoglobin A1C (HbA1c), fasting plasma glucose (FPG), 2 h postprandial plasma glucose (2 h PPG), albumin, creatinine, serum uric acid (SUA), C-reactive protein (CRP), total triglycerides (TG), total cholesterol (TC), low-density lipoprotein cholesterol (LDL-C), and high-density lipoprotein cholesterol (HDL-C) were measured by standard laboratory methods.

The 24 h urinary albumin excretion rate (UAER) was determined as urinary albumin output over a 24 h period. The urinary albumin-creatinine ratio (UACR) was estimated by a morning fasting spot urine sample. The estimated glomerular filtration rate (eGFR) was calculated using the Modification of Diet in Renal Disease (MDRD) Study formula: eGFR = 170 × (serum creatinine)^−0.999^ × (age)^−0.176^ × (serum urea nitrogen)^−0.170 ^× (serum albumin)^0.318^ × (0.762 females).

### 2.4. Echocardiography

Echocardiographic examination was carried out under two-dimensional guided M-mode with a Vingmed System 7 Doppler echocardiography unit (GE Vingmed Ultrasound, Philip, Germany) in patients with partial left lateral decubitus positions. Left ventricular mass (LVM) was calculated using the Devereux formula [15]: LVM  (g) = 0.8 × 1.04[(LVDD + PWTD + IVSD)^3^ − LVDD^3^] + 0.6. Body surface area (BSA) was calculated by the formula [16]: BSA  (m^2^) = 0.007184 × height  (cm)^0.725^ × weight  (kg)^0.425^. LVM index (LVMI) was derived by correcting LVM for BSA. LVH was defined as follows [17]: LVMI > 115 g/m^2^ for men and LVMI > 95 g/m^2^ for women.

### 2.5. Others

Type 2 diabetic kidney disease was diagnosed by the KDOQI guidelines in 2007 [18]. Alcohol consumption was defined as “ever drank” compared to “never drank.” Smoking was defined as “ever smoked” versus “never smoked.” Hypertension was defined as diastolic blood pressure (DBP) ≥ 90 mmHg and/or systolic blood pressure (SBP) ≥ 140 mmHg or current antihypertensive therapy. Dyslipidemia was defined as TG ≥ 2.26 mmol/L, HDL-C < 1.04 mmol/L, or LDL-C ≥ 4.14 mmol/L [19].

### 2.6. Statistical Analysis

Baseline characteristics were assessed with standard descriptive statistics. Data were expressed as either mean ± standard deviation. Different incontinuous variables were determined by independent samples *t*-test or one-way ANOVA. If data were not normally distributed or it did not meet the homogeneity of variances, nonparametric test was applied. The chi-square test was adopted for comparison of the prevalence data. A Spearman rank correlation test was utilised to assess the correlation between two variables. An ordinal logistic regression analysis was carried out for evaluation of SUA quartiles with the presence of LVH. Data analysis was performed by IBM SPSS statistics version 21.0 (SPSS, Chicago, IL). All tests were two sided, and a value of *p* < 0.05 was taken to be statistically significant.

## 3. Results

### 3.1. Clinical Characteristics

The numbers of subjects in this study were 282 men and 153 women with type 2 DKD. Overall, the mean level of eGFR was 52.45 (mL/min per 1.73 m^2^). Macroalbuminuria (UAER ≥ 300 mg/24 h) was 307 (70.6%). Left ventricular hypertrophy was observed in 153 (35.2%) patients.

The clinical characteristics of patients grouped by SUA quartiles have been illustrated in Table 1. The patients with higher SUA quartiles were more likely to be men. Moreover, percentage of the patients with HTN, CHD, and the use of AHAs was greater in patients who had higher SUA. There were no significant differences in age, smoking habits, alcohol consumption, duration of diabetes, SBP, DBP, and BMI among the SUA quartiles. Percentage of dyslipidemia, CI, and the use of LLDs was also not significantly different among SUA quartiles.

### 3.2. Laboratory and Echocardiographic Data

The laboratory and echocardiographic data of patients grouped by SUA quartiles is illustrated in Table 1. Significant differences were found among SUA quartiles in HbA1c, TG, HDL-C, eGFR, UAER, UACR, and echocardiographic data like LVDD, LVDS, IVST, and PWTD. As we all know that LVMI is a widely used method to assess LVH. Unusually, LVMI increased from 94.66 ± 28.31 g/m^2^ to 110.12 ± 30.08 g/m^2^ from SUA quartile 1 to quartile 4, and ratio of LVH increased from 25.9% to 44.4% from SUA quartile 1 to quartile 4 (Table 1).

### 3.3. Spearman's Rank Correlation Test

In the Spearman rank correlation test, LVMI showed significant positive correlation with SUA (*r* = 0.182, *p* < 0.001) and eGFR indicated significant negative correlation with SUA (*r* = −0.549, *p* < 0.001). Other parameters, such as UAER, UACR, and HbA1c, also had significant correlation with SUA (Table 2).

### 3.4. Logistic Regression Analysis

The logistic regression analysis (Table 3) shows association between SUA quartiles and the presence of LVH in type 2 diabetic kidney disease. After controlling of sex, age, smoking, alcohol, duration of diabetes, and the use of antihypertensive agents (model 1), compared with that with the subjects in quartile 1 (SUA < 324 *μ*mol/L), there was significant increased risk of LVH with the subjects in quartile 4 (SUA > 470 *μ*mol/L) (OR = 2.439; 95% CI 1.265–4.699; *p* = 0.008). The subjects in quartile 3 (SUA: 399–470 *μ*mol/L) also had a tendency to develop LVH compared with those in quartile 1 (OR = 1.593), but the *p* value was not significant (*p* = 0.183). After further controlling of SBP, DBP, HbA1c, TC, TG, HDL, and LDL (model 2), the risk of LVH with the subjects in quartile 4 remained increased significantly (OR = 2.576; 95% CI 1.150–5.768; *p* = 0.021). However, after further controlling of eGFR which is a recognized parameter that reflects the renal function and is significantly associated with SUA, there was no significant increased risk of LVH with the subjects in quartile 4 (OR = 1.750; 95% CI 0.685–4.470; *p* = 0.242) (model 3).

## 4. Discussion

The main finding of this study was that increased SUA level is positively associated with the increased risk of LVH in hospitalized Chinese patients with type 2 diabetic kidney disease, this association was independent of the effects of sex, age, smoking, alcohol, duration of diabetes, the use of antihypertensive agents, SBP, DBP, HbA1c, TC, TG, HDL, and LDL but was not independent of eGFR. This study includes a certain number of patients with DKD in stages 2–4 and macroalbuminuria. And the finding of this study could be generalized to the patients with the same characteristics.

Cardiovascular diseases are still the major cause of death among patients with type 2 DKD. LVH is a threatening prognostic sign and an independent risk factor for cardiovascular morbidity and mortality. A number of epidemiological studies have proved that LVH is common in patients with DKD. In the current study, there was also a high prevalence of LVH (35.2%) in patients with type 2 DKD.

The accumulated clinical and epidemiological studies have investigated the association between SUA and LVH, and the result remains unclear and debatable. Several studies have demonstrated that SUA level is an independent risk factor for LVH [8–12]. For example, a large Japanese clinical study that included 3305 male workers found out that the subjects with the highest SUA quartile exhibited a 1.58-fold increased risk for LVH (95% CI 1.23–2.02; *p* < 0.001) compared with those with the lowest SUA quartile [8]. In addition, Fujita et al. demonstrated that the association between SUA and LVH remained significant after adjustment for age, blood pressure, eGFR, and serum calcium-phosphate metabolism-related parameters Ca, phosphate, intact PTH, and FGF23 in male cardiac patients [11]. Hence, Catena et al. have suggested that SUA may play only an independent role in a specific population, such as women patients of hypertension. In that study, the multivariate regression analysis showed that the association between SUA and LVH was independent from components of the metabolic syndrome and renal function in women, but not in men [9]. Similarly, Yu et al. also found out that higher SUA level was significantly an independent risk factor for LVH in postmenopausal women (OR = 1.367; 95% CI 1.026–1.821), but not in premenopausal women (OR = 1.690; 95% CI 0.669–2.486) [10]. Some other studies confirmed the gender-related association between SUA and LVH [20, 21]. However, in several epidemiological studies, this positive association has failed to be confirmed [13, 14]. For example, a European clinical study which was conducted on 580 newly diagnosed, never treated, hypertensive patients with relatively low prevalence of hyperuricemia has reported no independent association between SUA and LVH [13]. Another large study arrived at the conclusion that SUA level was associated with microalbuminuria but not with LVH in the essential hypertensive subjects [14]. The associations between SUA level and the risk of LVH with special focus on type 2 DKD patients have not been investigated. Our own study found out that SUA level was associated with LVH, but this relationship was not independent of eGFR in type 2 diabetic kidney disease.

In this study, patients with higher SUA quartile had significantly lower level of eGFR (*p* < 0.001). The SUA level in the Spearman rank correlation test (*r* = −0.549; *p* < 0.001) was also negatively associated with eGFR. Thus, consistent with the previous study, we also found out that SUA level was significantly associated with eGFR. On the one hand, uric acid is predominantly excreted by the kidneys, and decline in eGFR will almost universally be associated with increased SUA [22]. On the other hand, uric acid could cause activation of RAAS, endothelial dysfunction, increase of oxidative stress, and inflammation in experimental models and thus lead to declining eGFR and tubulointerstitial fibrosis [4–6]. Moreover, the association between SUA and eGFR was also confirmed in several clinical studies [23–25].

As we have known, there was a two-way relationship between the kidney and the heart, namely, cardiorenal syndrome [26, 27]. A number of cross-sectional studies have concluded the reciprocation relationship between eGFR and LVH [28–30]. For example, a current clinical study which was conducted on 990 patients from Taiwan has concluded that eGFR might be an alternative method in risk stratification for increased LVH, and it was explained that patients who had low eGFR frequently faced electrolyte imbalance and volume retention which may lead to increased LVH and abnormal cardiac function [29]. However, our study also includes a certain number of patients with DKD in stages 2–4 and the mean eGFR was 52.45 (mL/min per 1.73 m^2^) of all the subjects.

Therefore, we suppose that uric acid may play an indirect role in the pathogenesis of LVH in type 2 diabetic kidney disease via eGFR which exhibited a significant correlation with SUA and LVH in the same population. For SUA and eGFR, which is chasing which? This is a problem that requires to be determined and investigated in further follow-up study.

There are several limitations for this study. First, we could not establish a causal relationship due to the cross-sectional studies. Second, there was certain heterogeneity among the subjects, although adequate adjustments have been made in the analysis of the study. A large-scale study and a further group analysis will be needed in future studies. Finally, a high-purine diet may induce elevation of SUA, and there was no information regarding the diet.

## 5. Conclusion

This study arrives at the conclusion that the elevated SUA level has positive association with the increased risk of LVH, but this relationship is not independent of eGFR which shows a significant correlation with SUA and LVH in the same population in hospitalized Chinese patients with type 2 diabetic kidney disease. Prospective studies are needed to determine the relationship of SUA level with eGFR and LVH. Therefore, whether decrease in SUA level in type 2 DKD patients by interventions may alleviate LVH awaits further investigation.

## Figures and Tables

**Table 1:** Clinical, biochemical, and echocardiographic characteristics of the study patients.

Variables	Q1 (*n* = 108)	Q2 (*n* = 109)	Q3 (*n* = 110)	Q4 (*n* = 108)	*p* value
Clinical characteristics					
SUA (*μ*mol/L)	<324	324–399	399–470	>470	—
Age (years)	64.4 ± 10.6	62.5 ± 11.1	63.5 ± 12.5	63.6 ± 13.7	0.687
Smoking (*n*, %)	20 (18.5%)	23 (21.1%)	24 (21.8%)	26 (24.1%)	0.846
Alcohol (*n*, %)	16 (14.8%)	19 (17.4%)	20 (18.2%)	21 (19.4%)	0.914
Male (*n*, %)	58 (53.7%)	69 (63.3%)	85 (77.3%)	70 (63.6%)	0.004
DD (years)	12.0 ± 6.6	12.8 ± 7.5	14.0 ± 8.4	12.5 ± 8.3	0.374
HTN (*n*, %)	66 (61.1%)	81 (74.3%)	91 (82.7%)	78 (72.2%)	<0.001
Dyslipidemia (*n*, %)	66 (61.1%)	80 (73.4%)	86 (78.2%)	79 (73.1%)	0.057
CI (*n*, %)	9 (8.3%)	10 (9.2%)	16 (14.5%)	15 (13.9%)	0.355
CHD (*n*, %)	12 (11.1%)	9 (8.3%)	19 (17.3%)	27 (25.0%)	0.015
AHAs (*n*, %)	59 (54.6%)	75 (68.8%)	79 (71.8%)	82 (75.9%)	0.006
LLDs (*n*, %)	6 (5.6%)	6 (5.5%)	6 (5.5%)	6 (5.6%)	0.985
SBP (mmHg)	141 ± 19	143 ± 23	142 ± 25	139 ± 22	0.683
DBP (mmHg)	81 ± 10	80 ± 13	82 ± 13	78 ± 13	0.230
BMI (kg/m^2^)	23.2 ± 3.2	24.8 ± 6.6	24.9 ± 3.5	25.3 ± 3.8	0.400
Biochemical variables					
FPG (mmol/L)	7.72 ± 2.93	7.61 ± 3.02	7.78 ± 3.67	7.47 ± 3.43	0.577
2 h PPG (mmol/L)	12.79 ± 4.61	12.23 ± 4.75	11.42 ± 4.68	11.61 ± 4.45	0.074
HbA1c (%)	8.6 ± 2.2	8.1 ± 1.9	7.5 ± 1.9	7.2 ± 1.7	<0.001
TC (mmol/L)	5.14 ± 1.67	5.19 ± 1.60	4.98 ± 1.64	5.13 ± 1.67	0.675
TG (mmol/L)	1.93 ± 2.26	1.96 ± 1.50	2.39 ± 1.79	1.76 ± 1.33	0.001
HDL-C (mmol/L)	1.16 ± 0.37	1.08 ± 0.30	1.01 ± 0.33	1.07 ± 0.38	0.003
LDL-C (mmol/L)	3.14 ± 1.43	3.10 ± 1.21	3.02 ± 1.11	2.95 ± 1.20	0.792
eGFR (mL/min per 1.73 m^2^)	86.1 ± 40.3	58.1 ± 39.9	41.6 ± 32.9	24.9 ± 24.6	<0.001
UAER (mg/24 h)	1247.5 ± 1721.7	2337.8 ± 2767.3	2362.8 ± 2521.7	2140.41 ± 1931.5	0.001
UACR (mg/g)	1270.9 ± 1984.6	2265.0 ± 2369.8	2309.2 ± 2224.4	2758.6 ± 2386.8	<0.001
CRP (mg/L)	8.15 ± 24.59	9.19 ± 22.61	7.65 ± 20.31	15.02 ± 23.64	0.246
Echocardiographic data					
LVDD (mm)	46.17 ± 3.99	46.50 ± 4.73	47.45 ± 5.48	48.82 ± 4.88	<0.001
LVDS (mm)	30.69 ± 4.27	31.47 ± 4.56	31.90 ± 4.59	33.19 ± 5.00	<0.001
IVST (mm)	10.35 ± 1.52	10.68 ± 1.84	11.08 ± 1.86	10.79 ± 1.73	0.028
PWTD (mm)	9.67 ± 2.23	9.93 ± 1.63	10.05 ± 1.64	10.06 ± 1.52	0.023
LVEF (%)	62 ± 5	61 ± 7	61 ± 5	60 ± 7	0.352
LVMI (g/m^2^)	94.66 ± 28.31	97.25 ± 31.28	103.38 ± 30.19	110.12 ± 30.08	<0.001
LVH (*n*, %)	28 (25.9%)	26 (23.9%)	35 (31.8%)	48 (44.4%)	0.004

SUA: serum uric acid; DD: duration of diabetes; HTN: hypertension; CI: cerebral infarction; CHD: coronary heart disease; AHAs: antihypertensive agents; LLDs: lipid-regulating drugs; SBP: systolic blood pressure; DBP: diastolic blood pressure; BMI: body mass index; FPG: fasting plasma glucose; 2 h PPG: 2 h postprandial plasma glucose; HbA1c: glycosylated hemoglobin A1c; TC: total cholesterol; TG: triglycerides; HDL-C: high-density lipoprotein cholesterol; LDL-C: low-density lipoprotein cholesterol; eGFR: estimated glomerular filtration; UAER: urinary albumin excretion rates; UACR: urinary albumin-creatinine ratio; CRP: C-reactive protein; LVDD: left ventricular end-diastolic dimension; LVDS: left ventricular end-systolic dimension; IVST: interventricular septal thickness; PWTD: posterior wall end-diastolic thickness; LVEF: left ventricular ejection fraction; LVMI: left ventricular mass index; LVH: left ventricular hypertrophy.

**Table 2:** Correlation coefficients between serum uric acid and various parameters.

	SUA (*μ*mol/L)	
	*r*	*p* value
SBP (mmHg)	−0.006	0.908
DBP (mmHg)	−0.072	0.147
BMI (kg/m^2^)	0.043	0.662
LVMI (g/m^2^)	0.182	<0.001
LVEF (%)	−0.065	0.188
HbA1c (%)	−0.241	<0.001
UAER (mg/24 h)	0.078	0.182
UACR (mg/g)	0.161	0.021
eGFR (mL/min per 1.73 m^2^)	−0.549	<0.001
TC (mmol/L)	−0.076	0.138
TG (mmol/L)	−0.029	0.571
HDL-C (mmol/L)	−0.104	0.043
LDL-C (mmol/L)	−0.071	0.171

SBP: systolic blood pressure; DBP: diastolic blood pressure; BMI: body mass index; LVMI: left ventricular mass index; LVEF: left ventricular ejection fraction; HbA1c: glycosylated hemoglobin A1c; UAER: urinary albumin excretion rates; UACR: urinary albumin-creatinine ratio; eGFR: estimated glomerular filtration; TC: total cholesterol; TG: triglycerides; HDL-C: high-density lipoprotein cholesterol; LDL-C: low-density lipoprotein cholesterol.

**Table 3:** Association of serum uric acid quartiles with LVH.

	ORs (95% CI), *p* value			
	Q1	Q2	Q3	Q4
*N*	108	109	110	108
Model 1	1 (reference)	0.837 (0.412–1.703), *p* = 0.624	1.593 (0.802–3.164), *p* = 0.183	2.439 (1.265–4.699), *p* = 0.008
Model 2	1 (reference)	0.701 (0.305–1.608), *p* = 0.401	1.990 (0.879–4.502), *p* = 0.099	2.576 (1.150–5.768), *p* = 0.021
Model 3	1 (reference)	0.625 (0.260–1.504), *p* = 0.294	1.342 (0.543–3.316), *p* = 0.523	1.750 (0.685–4.470), *p* = 0.242

Model 1: adjusted for sex, age, smoking, alcohol, duration of diabetes, and the use of antihypertensive agents; Model 2: further adjusted for SBP, DBP, HbA1c, TC, TG, HDL, and LDL; Model 3: further adjusted for eGFR; SBP: systolic blood pressure; DBP: diastolic blood pressure; TC: total cholesterol; TG: triglycerides; HDL-C: high-density lipoprotein cholesterol; LDL-C: low-density lipoprotein cholesterol; eGFR: estimated glomerular filtration.
